# Deaths from Chloroform

**Published:** 1897-12-04

**Authors:** 


### DEATHS FROM CHLOROFORM.

A very interesting and lucid address was recently delivered before the Society of Anæsthetists by their president, Dr. Dudley Buxton, in which he traced the history of anæsthetics from their introduction to the present time, and asked the question, Is the whole art and science of the medical man who practises in anæsthetics a matter of empiricism, or has he the right to claim for his calling the proud distinction of a science, and for his practice the equally aspiring title of a precise art? The answer to this is not altogether satisfactory, at least so far as progress in the general practice of anæsthesia is concerned. Dr. Buxton rightly said, "Syme's success in the use of chloroform is a matter of history, but his method was empiricism." The researches and conclusions of Snow, however, were scientific in the highest degree, and although we must fully recognise the immensity of the work which has been done since his time we ought with all humility to ask ourselves whether the ordinary teaching of anæsthetics, as now given in the schools, shows much, if any, advance upon the principles laid down by Snow. Are our students being taught empiricism or science when they are told to pour chloroform on a towel?

Dr. Buxton says, "Unhappily we cannot say the mortality as a whole is less," and, after all, this is the important point.

We have long thought that the fatality of anæsthesia was by no means fully recognised by the medical profession. We looked back through the bound volumes of our contemporaries and found but an imperfect record. Certain cases were given in full and interesting detail, but there was nothing to show the bulk of the mortality.

At the beginning of this year, therefore, we took measures to collect the records of all inquests on deaths from anæsthetics, so far as we were able to find them in the general press. On March 27th, we said, "We find that already in this year, not a quarter of which has yet passed, 22 deaths from chloroform have been recorded. It is a ghastly list of catastrophes to happen from the same cause in the course of three months in a country the size of England." On April 24th we recorded 5 more deaths from anæsthetics in the first 10 days of that month. On June 12th we gave notes of 12 more cases.

On August 14th we recorded 10 more cases, and now we have accumulated another list of 26; and we are by no means sure that we have got them all yet. At the least, then, we have to reckon with 74 deaths this year from anæsthetics in England. We will complete the series when the year is ended. A sad jubilee of a great discovery.

Is this the result of empiricism or of science? Dr. Dudley Buxton says: "A regrettable familiarity with the stoppered bottle and the always accessible towel or handkerchief has relaxed the sense of responsibility which once the giving of chloroform entailed." Again, he says: "The methods in vogue are usually faulty. The accomplished chloroformist will use the rudest means of bringing about chloroform sleep with impunity, but to leave the dose of anæsthetic and the degree of dilution to the judgment of the inexperienced house surgeon or dresser is to court disaster." Surely the facts we publish to-day are sufficient to show that all students should be taught a scientific rather than an empirical method for the administration of so potent and therefore so dangerous a drug. Yet medical men, who in all other matters are so accurate and careful, are still content to "spill" a little chloroform upon a towel when they wish to administer it to their patients.

